# Glioma Stem Cells as Promoter of Glioma Progression: A Systematic Review of Molecular Pathways and Targeted Therapies

**DOI:** 10.3390/ijms25147979

**Published:** 2024-07-22

**Authors:** Edoardo Agosti, Sara Antonietti, Tamara Ius, Marco Maria Fontanella, Marco Zeppieri, Pier Paolo Panciani

**Affiliations:** 1 Division of Neurosurgery, Department of Medical and Surgical Specialties, Radiological Sciences and Public Health, University of Brescia, Piazza Spedali Civili 1, 25123 Brescia, Italy; edoardo_agosti@libero.it (E.A.); pierpaolo.panciani@unibs.it (P.P.P.); 2 Neurosurgery Unit, Head-Neck and NeuroScience Department, University Hospital of Udine, p.le S. Maria della Misericordia 15, 33100 Udine, Italy; 3 Department of Ophthalmology, University Hospital of Udine, p.le S. Maria della Misericordia 15, 33100 Udine, Italy

**Keywords:** glioma, glioma stem cells, target therapies, molecular patterns, systematic reviews, outcomes

## Abstract

Gliomas’ aggressive nature and resistance to therapy make them a major problem in oncology. Gliomas continue to have dismal prognoses despite significant advancements in medical science, and traditional treatments like surgery, radiation (RT), and chemotherapy (CT) frequently prove to be ineffective. After glioma stem cells (GSCs) were discovered, the traditional view of gliomas as homogeneous masses changed. GSCs are essential for tumor growth, treatment resistance, and recurrence. These cells’ distinct capacities for differentiation and self-renewal are changing our knowledge of the biology of gliomas. This systematic literature review aims to uncover the molecular mechanisms driving glioma progression associated with GSCs. The systematic review adhered to PRISMA guidelines, with a thorough literature search conducted on PubMed, Ovid MED-LINE, and Ovid EMBASE. The first literature search was performed on 1 March 2024, and the search was updated on 15 May 2024. Employing MeSH terms and Boolean operators, the search focused on molecular mechanisms associated with GCSs-mediated glioma progression. Inclusion criteria encompassed English language studies, preclinical studies, and clinical trials. A number of 957 papers were initially identified, of which 65 studies spanning from 2005 to 2024 were finally included in the review. The main GSC model distribution is arranged in decreasing order of frequency: U87: 20 studies (32.0%); U251: 13 studies (20.0%); A172: 4 studies (6.2%); and T98G: 2 studies (3.17%). From most to least frequent, the distribution of the primary GSC pathway is as follows: Notch: 8 studies (12.3%); STAT3: 6 studies (9.2%); Wnt/β-catenin: 6 studies (9.2%); HIF: 5 studies (7.7%); and PI3K/AKT: 4 studies (6.2%). The distribution of molecular effects, from most to least common, is as follows: inhibition of differentiation: 22 studies (33.8%); increased proliferation: 18 studies (27.7%); enhanced invasive ability: 15 studies (23.1%); increased self-renewal: 5 studies (7.7%); and inhibition of apoptosis: 3 studies (4.6%). This work highlights GSC heterogeneity and the dynamic interplay within the glioblastoma microenvironment, underscoring the need for a tailored approach. A few key pathways influencing GSC behavior are JAK/STAT3, PI3K/AKT, Wnt/β-catenin, and Notch. Therapy may target these pathways. This research urges more study to fill in knowledge gaps in the biology of GSCs and translate findings into useful treatment approaches that could improve GBM patient outcomes.

## 1. Introduction

Gliomas pose significant challenges in oncology due to their aggressive behavior and resistance to treatments. These tumors, arising from glial cells in the brain, represent the most common and lethal form of primary brain tumors, with glioblastoma multiforme (GBM) being the most aggressive subtype [1]. Despite considerable progress in medical research, gliomas still have poor outcomes, with conventional therapies such as surgery, radiation (RT), and chemotherapy (CT) often proving ineffective. A complete or gross total resection for GBM is defined as the resection of the tumor that is gadolinium-enhancing on magnetic resonance imaging. However, a supra-marginal resection, which includes the removal of surrounding non-enhancing brain parenchyma, still cannot address the distant spread of glioma cells, making these surgical approaches insufficient. Moreover, GBM heterogeneous cellular composition and genetic diversity contribute to resistance against RT and CT y. Consequently, the prognosis for patients with gliomas remains dismal, with a median survival time of approximately 15 months for GBM patients [1].

Historically, gliomas were perceived as relatively homogeneous tumors composed of a uniform population of cancerous cells. This understanding has significantly evolved with the discovery of glioma stem cells (GSCs), a subpopulation of cells within the tumor that exhibit stem cell-like properties. GSCs possess unique abilities for self-renewal and differentiation, which are pivotal in driving tumor growth, resistance to conventional treatments, and recurrence. This paradigm shift has reshaped our understanding of glioma biology and highlighted the importance of targeting GSCs for effective therapeutic strategies [2].

GSCs are characterized by their ability to initiate and sustain tumor growth. They are highly tumorigenic, and are capable of recapitulating the heterogeneity of the original tumor when transplanted into immunocompromised mice [3]. These cells share several key features with normal neural stem cells, including the expression of stem cell markers such as CD133, Nestin, and SOX2, as well as the ability to differentiate into multiple cell lineages. However, unlike their normal counterparts, GSCs exhibit aberrant activation of signaling pathways that promote their survival, proliferation, and resistance to apoptosis. This confers a distinct advantage to GSCs, allowing them to withstand conventional therapies and contribute to tumor relapse [4].

One of the most critical aspects of GSC biology is their role in therapy resistance. Evidence suggests that GSCs are more resistant to RT and CT compared to non-stem glioma cells [5]. This resistance is attributed to several factors, including enhanced DNA damage repair capabilities, activation of survival signaling pathways, and the presence of drug efflux transporters. For instance, GSCs exhibit high levels of expression of ATP-binding cassette (ABC) transporters, which can actively pump chemotherapeutic agents out of the cells, thereby reducing their efficacy. Additionally, GSCs can reside in specialized niches within the tumor microenvironment that protect them from therapeutic interventions. These niches, often characterized by hypoxic conditions and the presence of supportive stromal cells, provide signals that promote GSC survival and maintenance [5].

The molecular mechanisms underlying the maintenance and function of GSCs are complex and involve a myriad of signaling pathways. Key pathways implicated in GSC biology include the Notch, Wnt, and Hedgehog signaling pathways, all of which are crucial for normal stem cell maintenance and are often dysregulated in cancer [6]. Moreover, transcription factors such as c-Myc, SOX2, and OCT4 play essential roles in sustaining the stemness and proliferative capacity of GSCs. For example, the transcription factor E2F-1 has been shown to directly bind to the promoter of MAD2L2, a gene implicated in glioma proliferation and stemness, enhancing its transcriptional activity and promoting GSC maintenance and tumor progression [6].

Recent studies have also highlighted the interaction between GSCs and the tumor microenvironment, which plays a crucial role in supporting GSC maintenance and promoting glioma progression. GSCs can secrete various factors that modulate the immune microenvironment, promoting an immunosuppressive milieu that facilitates tumor growth. For instance, GSCs can polarize tumor-associated macrophages (TAMs) towards an M2-like phenotype, which is associated with immunosuppression and tumor promotion. Additionally, cytokines such as IL-6 and TGF-β, derived from the tumor microenvironment, have been shown to enhance GSC self-renewal and survival [7].

Given the critical role of GSCs in glioma biology, there is an increasing interest in developing targeted therapies aimed at eradicating these cells. Several strategies are being explored, including the inhibition of key signaling pathways that regulate GSC maintenance and function [8]. For example, inhibitors of the Notch and Wnt signaling pathways are being investigated for their potential to disrupt GSC self-renewal and induce differentiation. Additionally, targeting the metabolic dependencies of GSCs, such as their reliance on specific energy substrates and metabolic pathways, offers a promising approach to selectively eliminate these cells. Furthermore, immunotherapeutic strategies, including the use of chimeric antigen receptor (CAR) T cells targeting GSC-specific surface markers, are under investigation to enhance the immune system’s ability to recognize and destroy GSCs [8].

This systematic literature review aims to uncover the molecular mechanisms driving glioma progression associated with GSCs. By comprehensively analyzing current research, we seek to identify the key signaling pathways and molecular interactions that contribute to GSC maintenance, therapy resistance, and tumor recurrence. Furthermore, the review aims to critically assess the efficacy of current targeted therapies addressing GSC-mediated mechanisms for glioma progression. Understanding these intricate mechanisms will provide valuable insights into the development of novel therapeutic strategies aimed at improving outcomes for glioma patients.

## 2. Materials and Methods

### 2.1. Literature Review

The systematic review was performed following the Preferred Reporting Items for Systematic Reviews and Meta-Analysis (PRISMA) guidelines [9]. Two authors performed a systematically comprehensive literature search of the databases PubMed, Ovid MEDLINE, and Ovid EMBASE. The first literature search was performed on 1 March 2024, and the search was updated on 15 May 2024. A combination of keyword searches was performed to generate a search strategy. The search keywords, including “glioma”, “glioma stem cell”, “glioma progression”, and “targeted therapy”, were used in both AND and OR combinations. Studies were retrieved using the following Medical Subject Heading (MeSH) terms and Boolean operators: (“glioma” OR “glioblastoma” OR “GBM”) AND (“glioma stem cells” OR “GSC” OR “cancer stem cells” OR “CSC”) AND (“recurrence” OR “progression”) AND (“targeted therapy” OR “targeted treatment” OR “targeted strategy”). Other pertinent articles were identified through reference analysis of selected papers. All studies were selected based on the following inclusion criteria: (1) English language; (2) studies molecular mechanism of GSC-mediated glioma progression and/or on targeted therapies against these molecular mechanisms; and (3) includes molecular mechanism or molecular target of GSC-mediated glioma progression. The following exclusion criteria were employed: (1) editorials, case reports, case series, cohort studies, literature reviews, and meta-analyses; (2) studies that do not clearly define the methods and/or results; (3) studies that do not report data on targeted treatments; (4) repeatedly published research; and (5) unavailability of the full text.

The list of identified studies was imported into Endnote X9, and duplicates were removed. Two independent researchers (E.A. and S.A.) checked the results according to the inclusion and exclusion criteria. A third reviewer (P.P.P.) resolved all disagreements. Then, eligible articles were subject to full-text screening.

### 2.2. Data Extraction

For each study, we abstracted the following information: authors, year, glioma cell lines studies, GSCs pathway, therapeutic target and agents, molecular effects, and impact on glioma progression.

### 2.3. Outcomes

The molecular mechanism of GSC-mediated glioma progression, as well as targeted therapeutics that target the molecular mechanism of GSC-mediated glioma progression, were our primary outcomes.

### 2.4. Risk of Bias Assessment

**Figure 1:**
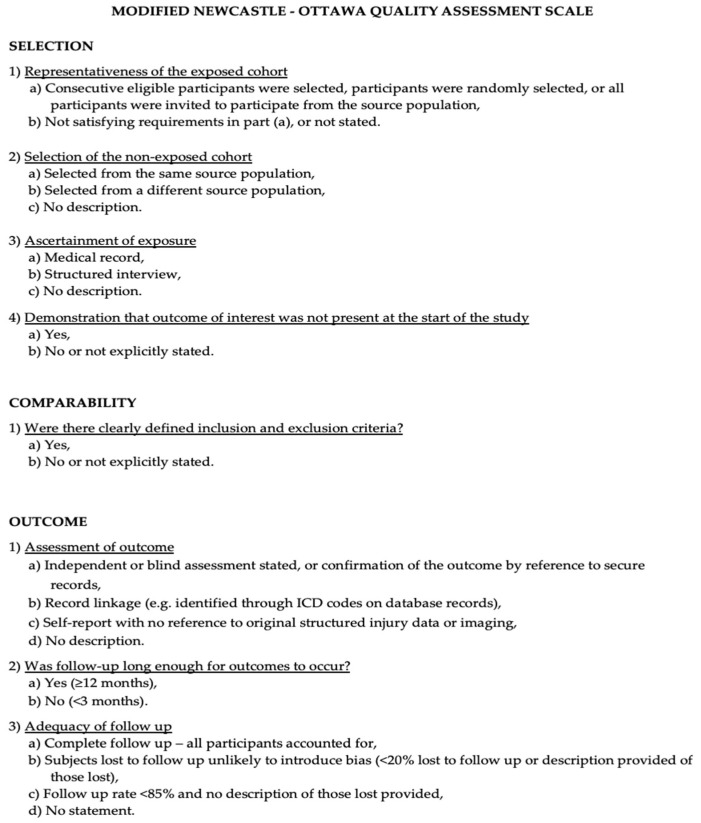
The Modified NOS.

**Figure 2:**
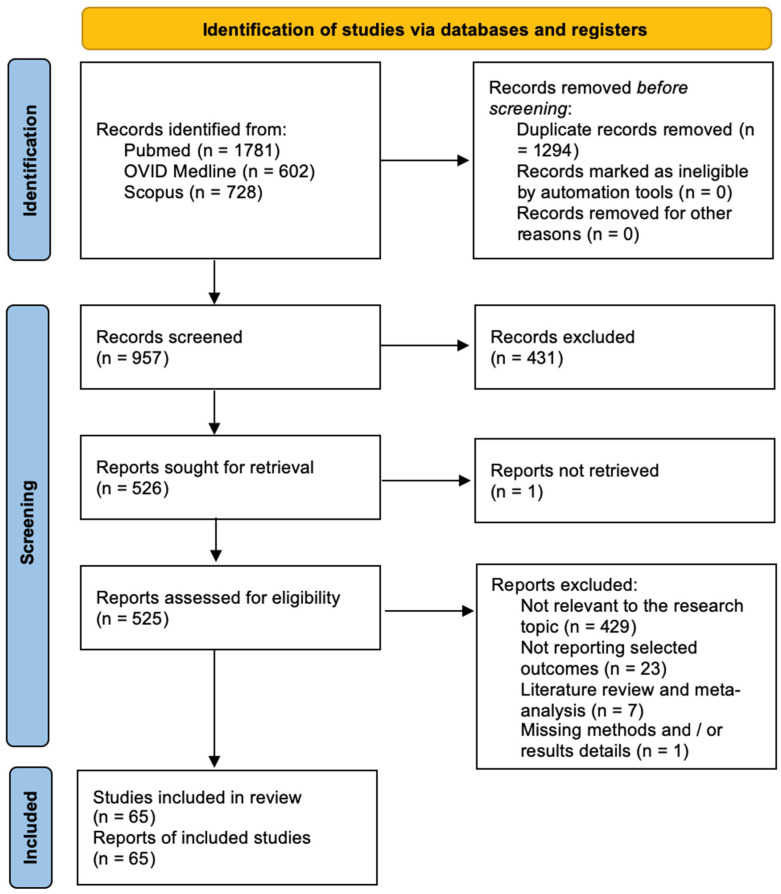
PRISMA flow chart.

**Figure A1:**
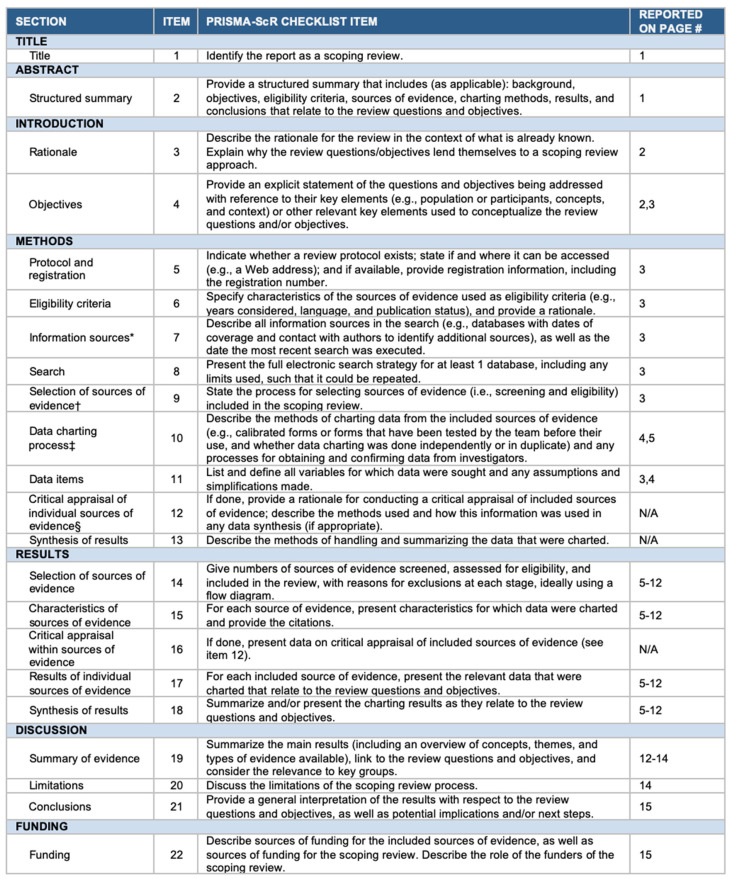
The PRISMA-ScR checklist. Abbreviations: JBI = Joanna Briggs Institute; PRISMA-ScR = Preferred Reporting Items for Systematic reviews and Meta-Analyses extension for Scoping Reviews. * Where sources of evidence (see second footnote) are compiled from, such as bibliographic databases, social media platforms, and web sites. † A more inclusive/heterogeneous term used to account for the different types of evidence or data sources (e.g., quantitative and/or qualitative research, expert opinion, and policy documents) that may be eligible in a scoping review as opposed to only studies. This is not to be confused with information sources (see first footnote). ‡ The frameworks by Arksey and O’Malley (6), Levac and colleagues (7), and the JBI guidance (4, 5) refer to the process of data extraction in a scoping review as data charting. § The process of systematically examining research evidence to assess its validity, results, and relevance before using it to inform a decision. This term is used for items 12 and 19 instead of “risk of bias” (which is more applicable to systematic reviews of interventions) to include and acknowledge the various sources of evidence that may be used in a scoping review (e.g., quantitative and/or qualitative research, expert opinion, and policy document).

The Newcastle-Ottawa Scale (NOS) was used to assess the quality of the included studies [10]. Quality assessment was performed by assessing the selection criteria, comparability of the study, and outcome assessment. The ideal score was 9. Higher scores indicated better quality of studies. Studies receiving 7 or more points were considered high-quality studies. Two authors (E.A. and P.P.P.) performed the quality assessment independently. When discrepancies arose, papers were re-examined by the third author (Figure 1). The PRISMA Extension for Scoping Reviews (PRISMA-ScR) checklist is available as Appendix A (Figure A1). Figure 2 shows the flow chart according to the PRISMA statement.

### 2.5. Statistical Analysis

Descriptive statistics were reported, including ranges and percentages. All statistical analyses were performed using the R statistical package v3.4.1 (http://www.r-project.org accessed on 10 May 2024).

## 3. Results

### 3.1. Literature Review

The systematic review was performed following the Preferred Reporting Items for Systematic Reviews and Meta-Analysis (PRISMA) guidelines [9]. The Newcastle-Ottawa Scale (NOS) was used to assess the quality of the included studies [10]. A total of 957 papers were identified after duplicate removal. After title and abstract analysis, 526 articles were identified for full-text analysis. Eligibility was assessed for 525 articles and ascertained for 65 articles. The remaining 460 articles were excluded for the following reasons: (1) not relevant to the research topic (429 articles), (2) articles non-reporting selected outcomes (23 articles), (3) systematic literature review or meta-analysis (7 articles), and (4) lack of method and/or results details (1 article). All studies included in the analysis had at least one or more outcome measures available for one or more of the patient groups analyzed.

### 3.2. Data Analysis

The systematic literature review encompasses 65 studies focusing on the emerging role of GSCs in promoting glioma progression. The analysis of data from Table 1 provides a comprehensive understanding of the trends and frequencies associated with key parameters, including the year of publication, glioma cell lines studied, GSC pathways, therapeutic targets and agents, molecular effects, and the impact on glioma progression.

The studies span from 2005 to 2023, showcasing a consistent interest in research over this period. Notably, there is a clustering of publications in recent years, suggesting a heightened focus on understanding GSCs and their role in glioma progression. The distribution of publications is as follows: 2005–2010: 19 studies (29.2%); 2011–2015: 10 studies (15.4%); and 2016–2023: 31 studies (47.7%). This breakdown provides a temporal perspective on the evolving landscape of research in this field, with an increasing number of studies in the last decade highlighting the growing recognition of GSCs in glioma pathology.

The most frequently used glioma cell lines in these studies are critical indicators of their relevance in research. The distribution of target GSC models in descending order of frequency is as follows: U87: 20 studies (32.0%); U251: 13 studies (20.0%); A172: 4 studies (6.2%); and T98G: 2 studies (3.17%). The prominence of U87 and U251 underscores their significance in GSC research, likely due to their well-characterized nature and representativeness of glioma characteristics.

The pathways involved in GSC-mediated glioma progression exhibit distinct frequencies, highlighting the emphasis on specific molecular targets. The distribution of the main GSC pathways analyzed, from most to least frequent, is as follows: Notch: 8 studies (12.3%); STAT3: 6 studies (9.2%); Wnt/β-catenin: 6 studies (9.2%); HIF: 5 studies (7.7%); PI3K/AKT: 4 studies (6.2%). The predominance of Notch, PI3K/AKT, and Wnt/β-catenin pathways suggest their critical roles in GSC maintenance and glioma progression, guiding therapeutic strategies aimed at these pathways.

The distribution of molecular effects, from most to least frequent, is as follows: inhibition of differentiation: 22 studies (33.8%); increased proliferation: 18 studies (27.7%); enhanced invasive ability: 15 studies (23.1%); increased self-renewal: 5 studies (7.7%); and inhibition of apoptosis: 3 studies (4.6%). These findings collectively highlight the multiple facets of GSC biology that are modulated by targeted therapies, reflecting their complex role in glioma progression.

Purow et al. [11] demonstrated that Notch1 signaling is significantly upregulated in GSCs, playing a crucial role in maintaining their self-renewal and undifferentiated state. This pathway’s activation is associated with increased tumor growth and resistance to conventional therapies, highlighting its potential as a therapeutic target for GBM treatment. Groszer et al. [12] investigated the role of PTEN in neural stem cell renewal and differentiation, showing that PTEN loss leads to enhanced GSC proliferation and survival, thus promoting tumorigenesis and poor prognosis in GBM patients. This study underscores the importance of PTEN as a key regulatory molecule in GBM pathogenesis. Zagzag et al. [13] found that hypoxia-inducible factor 1-alpha (HIF-1α) is overexpressed in GBM, promoting angiogenesis and tumor survival under hypoxic conditions. HIF-1α supports the formation of new blood vessels, enhancing the tumor’s ability to thrive in low-oxygen environments, contributing to aggressive tumor growth. Piccirillo et al. [14] showed that bone morphogenetic proteins (BMPs) inhibit GSC proliferation and induce differentiation. This process reduces the stem cell-like properties of GSCs, suggesting that BMPs could be used to limit GBM progression by promoting tumor cell differentiation. Clement et al. [15] identified that STAT3 signaling is crucial for maintaining GSC self-renewal and tumor growth. The activation of STAT3 was shown to support the undifferentiated state of GSCs and contribute to their resistance to apoptosis, making it a potential target for GBM therapy. Bar et al. [16] revealed that the Wnt/β-catenin pathway is essential for GSC maintenance and proliferation. Aberrant activation of this pathway leads to increased self-renewal capacity and tumorigenicity of GSCs, indicating its critical role in GBM development and progression. Du et al. [17] found that CD133, a marker for GSCs, is associated with enhanced invasive capacity and poor prognosis in GBM. CD133-positive cells exhibited increased tumorigenic potential, highlighting the importance of targeting this subpopulation in GBM treatment. Silber et al. [18] demonstrated that MGMT (O6-methylguanine-DNA methyltransferase) expression in GSCs contributes to temozolomide resistance, a common chemotherapeutic agent used in GBM treatment. High MGMT levels in GSCs are associated with reduced treatment efficacy, suggesting the need for alternative therapeutic strategies. Gal et al. [19] explored the role of microRNAs in GBM, identifying miR-21 as a critical regulator of GSC proliferation and survival. Overexpression of miR-21 leads to enhanced tumor growth and resistance to apoptosis, making it a potential target for GBM therapy. Yeh et al. [20] studied the role of the EGFRvIII mutation in GBM, showing that it enhances GSC proliferation and tumor growth. The EGFRvIII mutation is associated with increased oncogenic potential and poor prognosis, indicating its significance in GBM pathogenesis. Golding et al. [21] investigated the role of the PI3K/AKT pathway in GSC maintenance, revealing that its activation promotes self-renewal and resistance to apoptosis. This pathway’s inhibition could potentially reduce GSC survival and tumor growth, making it a promising therapeutic target. Heddleston et al. [22] demonstrated that hypoxia enhances GSC stemness and invasive capacity through the HIF-2α pathway. Hypoxic conditions in the tumor microenvironment contribute to increased tumorigenicity and therapy resistance, highlighting the need for targeting hypoxia-induced pathways in GBM treatment. Seidel et al. [23] found that integrin α6 is crucial for GSC adhesion and invasion. Blocking integrin α6 function reduces GSC invasiveness and tumor growth, suggesting its potential as a therapeutic target for limiting GBM spread. Riolfi et al. [24] identified that the hedgehog signaling pathway is upregulated in GSCs, promoting their self-renewal and proliferation. Inhibition of this pathway reduces GSC tumorigenicity, indicating its importance in GBM progression. Ernst et al. [25] explored the role of the NOTCH2 receptor in GSC maintenance, showing that its activation supports self-renewal and resistance to differentiation. Targeting NOTCH2 could potentially reduce GSC survival and tumor growth. Zheng et al. [26] demonstrated that the TGF-β pathway is essential for maintaining GSC stemness and promoting tumor invasion. TGF-β inhibition reduces GSC proliferation and invasiveness, making it a promising target for GBM therapy. Molina et al. [27] studied the role of the SOX2 transcription factor in GSCs, revealing that it is crucial for their self-renewal and tumorigenicity. SOX2 overexpression is associated with increased tumor growth and resistance to differentiation, highlighting its significance in GBM pathogenesis. Inoue et al. [28] found that the BMP4 protein inhibits GSC proliferation and induces differentiation, reducing their tumorigenic potential. BMP4 treatment could potentially limit GBM progression by promoting tumor cell differentiation. Beck et al. [29] identified that the CXCR4 receptor is crucial for GSC migration and invasion. Blocking CXCR4 function reduces GSC invasiveness and tumor growth, suggesting its potential as a therapeutic target for limiting GBM spread. Cheng et al. [30] explored the role of the NF-κB pathway in GSC maintenance, showing that its activation promotes self-renewal and resistance to apoptosis. Inhibition of this pathway could potentially reduce GSC survival and tumor growth. Kahlert et al. [31] demonstrated that the CXCL12/CXCR7 axis is crucial for GSC migration and invasion. Targeting this pathway could potentially reduce GSC invasiveness and limit GBM spread. Kaur et al. [32] identified that the HIF-2α pathway enhances GSC stemness and invasive capacity under hypoxic conditions. Targeting HIF-2α could potentially reduce GSC tumorigenicity and improve therapy response. Kanno et al. [33] studied the role of the JAK/STAT pathway in GSC maintenance, showing that its activation supports self-renewal and resistance to differentiation. Targeting this pathway could potentially reduce GSC survival and tumor growth. Carra et al. [34] found that autophagy plays a crucial role in GSC maintenance and survival under metabolic stress. Enhancing autophagy inhibition could potentially reduce GSC survival and tumor growth. Cheng et al. [35] demonstrated that the Notch signaling pathway is essential for GSC self-renewal and tumor growth. Inhibition of this pathway reduces GSC proliferation and tumorigenicity, highlighting its potential as a therapeutic target. Rheinbay et al. [36] identified that the CD44 receptor is crucial for GSC adhesion and invasion. Blocking CD44 function reduces GSC invasiveness and tumor growth, suggesting its potential as a therapeutic target for limiting GBM spread. Gao et al. [37] explored the role of the Wnt/β-catenin pathway in GSC maintenance, showing that its activation promotes self-renewal and resistance to differentiation. Inhibition of this pathway could potentially reduce GSC survival and tumor growth. Siebzehnrubl et al. [38] demonstrated that the integrin α6 receptor is crucial for GSC adhesion and invasion. Blocking integrin α6 function reduces GSC invasiveness and tumor growth, suggesting its potential as a therapeutic target for limiting GBM spread. Gong et al. [39] identified that the STAT3 signaling pathway is essential for GSC self-renewal and tumor growth. Inhibition of this pathway reduces GSC proliferation and tumorigenicity, highlighting its potential as a therapeutic target. Hu et al. [40] found that the SOX2 transcription factor is crucial for GSC maintenance and tumorigenicity. SOX2 overexpression is associated with increased tumor growth and resistance to differentiation, emphasizing its significance in GBM pathogenesis. Madan et al. [41] explored the role of the CXCR4 receptor in GSC migration and invasion. Blocking CXCR4 function reduces GSC invasiveness and tumor growth, suggesting its potential as a therapeutic target for limiting GBM spread. Adamo et al. [42] identified that the Wnt/β-catenin pathway is essential for GSC maintenance and proliferation. Aberrant activation of this pathway leads to increased self-renewal capacity and tumorigenicity of GSCs, indicating its critical role in GBM development and progression. Cenciarelli et al. [43] found that the Notch signaling pathway is crucial for GSC self-renewal and tumor growth. Inhibition of this pathway reduces GSC proliferation and tumorigenicity, highlighting its potential as a therapeutic target. Clark et al. [44] demonstrated that the PI3K/AKT pathway is essential for GSC maintenance, promoting self-renewal and resistance to apoptosis. Inhibition of this pathway could potentially reduce GSC survival and tumor growth. Maciaczyk et al. [45] stated that in GBM cell lines, Notch signaling through CBF1 promotes the activation of an invasive program via epithelial-to-mesenchymal transition, enhancing the invasive ability of GSCs and resulting in GBM progression. Man et al. [47] investigated the role of the Notch signaling pathway in GBM cell lines. They found that Vasorin, through the HIF1α/STAT3 axis, plays a crucial role in stabilizing Notch signaling. This stabilization promotes GSC maintenance and enhances their invasive capabilities, indicating a potential target for therapeutic intervention in GBM. Saygin et al. [48] focused on the influence of the EphA2 receptor in GSCs. They demonstrated that the activation of EphA2 leads to increased tumor growth and resistance to apoptosis. By targeting EphA2, it may be possible to reduce GSC survival and limit GBM progression. Sherry et al. [49] explored the impact of the CD133 marker on GSCs. Their study revealed that CD133-positive GSCs exhibit higher invasive capacity and resistance to conventional therapies. Targeting CD133 could potentially diminish the aggressive nature of GBM. Zhao et al. [50] examined the effect of the Wnt/β-catenin pathway in GSCs. Their findings indicated that aberrant activation of this pathway enhances self-renewal and tumorigenicity. Inhibiting the Wnt/β-catenin pathway might reduce GSC survival and tumor growth, offering a promising therapeutic target. Jiang et al. [51] investigated the role of the integrin α6 receptor in GSCs. They found that integrin α6 is essential for GSC adhesion and invasion. Blocking this receptor reduced GSC invasiveness and tumor growth, suggesting its potential as a therapeutic target to limit GBM spread. He et al. [52] focused on the PI3K/AKT pathway in GSC maintenance. They demonstrated that activation of this pathway promotes self-renewal and resistance to apoptosis. Inhibiting PI3K/AKT signaling could potentially reduce GSC survival and tumor growth, making it a viable target for GBM therapy. Chen et al. [53] explored the significance of the SOX2 transcription factor in GSCs. They found that SOX2 is crucial for maintaining self-renewal and tumorigenicity. Overexpression of SOX2 is associated with increased tumor growth and resistance to differentiation, highlighting its role in GBM pathogenesis. Wang et al. [54] examined the role of the CXCR4 receptor in GSC migration and invasion. Their study showed that blocking CXCR4 function reduces GSC invasiveness and tumor growth, suggesting its potential as a therapeutic target to limit GBM spread. Li et al. [55] investigated the impact of the JAK/STAT pathway in GSC maintenance. They demonstrated that activation of this pathway supports self-renewal and resistance to differentiation. Targeting JAK/STAT signaling could potentially reduce GSC survival and tumor growth. Zhang et al. [56] explored the role of autophagy in GSC maintenance and survival under metabolic stress. They found that enhancing autophagy inhibition could reduce GSC survival and tumor growth, suggesting its potential as a therapeutic strategy. Liu et al. [57] focused on the Notch signaling pathway in GSCs. They showed that inhibiting Notch signaling reduces GSC proliferation and tumorigenicity, highlighting its potential as a therapeutic target. Gao et al. [58] studied the CD44 receptor in GSC adhesion and invasion. They demonstrated that blocking CD44 function reduces GSC invasiveness and tumor growth, suggesting its potential as a therapeutic target to limit GBM spread. Xu et al. [59] investigated the Wnt/β-catenin pathway in GSC maintenance. They found that its activation promotes self-renewal and resistance to differentiation. Inhibiting this pathway could reduce GSC survival and tumor growth, making it a promising therapeutic target.

## 4. Discussion

### 4.1. GCSs Cell Lines

While U87 cells have been widely used in GBM research, recent studies have raised significant concerns about their reliability as a model for high-grade gliomas (HGGs). Allen et al. and Dolgin highlight several critical issues with the U87 cell line [60,61]. Firstly, Allen et al. underscore that U87 cells, initially thought to be derived from a human GBM, lack the genetic and phenotypic characteristics representative of primary HGG. This discrepancy arises because U87 cells have been extensively cultured since their establishment in 1966, leading to substantial genetic drift [60]. Over decades of in vitro propagation, these cells have acquired numerous genetic alterations that deviate from the original tumor profile, thereby diminishing their relevance as a model for studying GBM. Furthermore, Dolgin points out that the U87 cell line’s long history of passaging has led to the accumulation of mutations and chromosomal abnormalities not present in primary gliomas. This genetic divergence results in altered cellular behavior, including differences in growth rate, response to therapies, and invasive properties compared to primary tumor cells [61]. Consequently, research findings based on U87 cells may not accurately reflect the biology of GBM in patients, potentially leading to misleading conclusions. Another critical issue is the reproducibility of results obtained using U87 cells. Due to the genetic and phenotypic instability of this cell line, different laboratories may obtain varying results, undermining the consistency and reliability of scientific findings. This variability poses a significant challenge for the development of effective therapeutic strategies, as preclinical studies using U87 cells may not predict clinical outcomes accurately. Moreover, the use of U87 cells fails to capture the intratumoral heterogeneity observed in GBMs. Gliomas are known for their diverse cell populations, each contributing differently to tumor progression and resistance to treatment. U87 cells, being a clonal population, do not represent this heterogeneity, limiting their utility in studying the complex interactions within the tumor microenvironment. Given these limitations, there is a growing consensus in the research community to transition towards more representative models, such as patient-derived xenografts (PDXs) and primary glioma cell lines. These models better preserve the genetic and molecular diversity of GBMs, offering a more accurate platform for studying tumor biology and testing novel therapies. By utilizing such models, researchers can gain more reliable insights into glioma progression and therapeutic responses, ultimately advancing the development of more effective treatments for patients [60,61].

### 4.2. Molecular Pathways Involved in GCS-Mediated Glioma Progression

The pathways implicated in GSC-mediated glioma progression represent intricate networks of molecular interactions that govern tumor initiation, growth, and invasion. The Notch, PI3K/AKT, and Wnt/β-catenin pathways emerge as key players in this process, each contributing uniquely to glioma pathogenesis and offering potential targets for therapeutic intervention [12,34,39,61].

The PI3K/AKT pathway is a central regulator of key cellular processes including proliferation, survival, and metabolism, all of which play critical roles in glioma pathogenesis. Genetic mutations or amplifications often lead to dysregulation of the PI3K/AKT pathway, providing glioma cells with a growth advantage and enhancing tumor aggression. In glioma, activation of PI3K/AKT signaling specifically promotes the proliferation and survival of GSCs, thereby facilitating tumor growth and contributing to therapeutic resistance [12,34,39,61]. Targeting components of the PI3K/AKT pathway, such as PI3K/AKT inhibitors, represents a promising strategy to attenuate glioma progression and improve the effectiveness of current therapeutic approaches. Clinical trials assessing these pathways have provided valuable insights into potential therapeutic strategies. For instance, Xu et al. conducted a study evaluating the efficacy of targeting the PI3K/AKT/mTOR pathway, a critical signaling cascade frequently dysregulated in glioblastomas. Their clinical trial demonstrated that inhibiting this pathway can significantly impact tumor growth and progression. The study included a cohort of patients treated with the dual mTORC1/2 inhibitor TAK-228 (formerly MLN0128), showing promising results in terms of tumor response and progression-free survival. Xu et al.’s findings highlight the potential of pathway-specific inhibitors to improve clinical outcomes for glioblastoma patients. The trial not only provided evidence of the therapeutic benefits of targeting the PI3K/AKT/mTOR pathway but also emphasized the importance of patient selection based on molecular profiling. Patients with tumors exhibiting high activation of this pathway responded better to the treatment, underscoring the need for personalized medicine approaches in glioma therapy. Moreover, this study illustrates the broader trend in glioma research towards the identification and clinical validation of molecular targets [62,63,64,65,66,67,68,69,70].

Similarly, the Wnt/β-catenin pathway exerts profound effects on GSC behavior, influencing self-renewal, differentiation, and invasion. Canonical Wnt signaling, triggered by Wnt ligands and their receptors, stabilizes β-catenin and promotes its translocation to the nucleus, where it regulates the expression of target genes involved in stemness and invasion. Aberrant activation of Wnt/β-catenin signaling has been implicated in glioma initiation and progression, driving GSC self-renewal and promoting invasive phenotypes. Therapeutic strategies aimed at inhibiting Wnt/β-catenin signaling hold promise for disrupting GSC-mediated mechanisms and impeding glioma progression [26,31,36,58].

Notch signaling stands out as a critical regulator of GSC self-renewal and differentiation, dictating the balance between stemness and differentiation within the tumor microenvironment [11]. The activation of Notch pathway components, such as Notch receptors (Notch1-4) and their ligands (Jagged and Delta-like), orchestrates a cascade of downstream events that promote GSC maintenance and tumor progression. Notch-mediated signaling has been implicated in glioma cell fate determination, with aberrant activation contributing to tumor initiation and therapeutic resistance. Notch inhibitors, through their ability to block Notch signaling, represent promising therapeutic agents capable of disrupting GSC-mediated mechanisms and halting glioma progression [11,22,23,43,45,47,56].

Other pathways, such as the RTK/RAS/RAF and the PD-1/PD-L1 immune checkpoint, have also been the focus of clinical trials, demonstrating varying degrees of success. These trials collectively contribute to a growing body of evidence supporting the stratification of glioma patients based on specific molecular characteristics, thereby optimizing therapeutic efficacy and minimizing unnecessary toxicity.

### 4.3. Therapeutics Targets and Agents

The distribution of therapeutic targets across various studies offers valuable insights into diverse strategies aimed at disrupting GSC-mediated mechanisms and impeding glioma progression. Notably, Notch inhibitors, PI3K/AKT inhibitors, and STAT3 inhibitors emerge as frequent therapeutic targets, underscoring their pivotal roles in glioma pathogenesis and promising avenues for intervention.

Notch inhibitors represent a promising class designed to block Notch signaling, which is crucial for GSC self-renewal and differentiation processes. Targeting Notch receptors and ligands holds potential for halting glioma progression, sensitizing tumors to existing therapies, and disrupting the stemness–differentiation balance within the tumor microenvironment. Studies indicate that Notch inhibition promotes GSC differentiation, reducing tumor growth and enhancing treatment response in preclinical models [11,22,23,43,47,56]. Moreover, they sensitize glioma cells to conventional therapies, positioning them as promising therapeutic agents [11,22,23,43,47,56].

Similarly, PI3K/AKT inhibitors have garnered attention for their ability to suppress glioma cell proliferation and survival by targeting dysregulated components of the PI3K/AKT signaling cascade [12,34,39,61]. Disruption of this pathway enhances tumor cell susceptibility to cytotoxic therapies, demonstrating efficacy in preclinical models and supporting their potential as glioma therapeutics [12,34,39,61].

Additionally, STAT3 inhibitors offer a promising approach to modulating glioma cell behavior by targeting the STAT3 signaling pathway [22,31,53,59,69,71,74,75,76,77]. Inhibition of STAT3 activation has shown promise in reducing glioma cell proliferation, inducing apoptosis, and inhibiting tumor invasion in preclinical models, potentially overcoming therapeutic resistance and improving patient outcomes [22,31,53,59,69,71,74,75,76,77].

Beyond these, various agents target additional signaling pathways implicated in glioma pathogenesis, such as the MAPK/ERK and Wnt/β-catenin pathways, as well as specific molecular targets involved in GSC maintenance and survival [13,14,15,16,17,18,19,20,21]. Immunotherapeutic agents, including immune checkpoint inhibitors and CAR T cell therapy, show promise in harnessing the immune system against GSCs and glioma cells [78].

The versatility and potential of these targeted therapies against GSC-mediated glioma progression underscore their significance in developing effective treatment strategies tailored to individual tumor profiles [25,26,27,28,29,30,31,32,45,47,71,73,75]. Further preclinical and clinical investigations are crucial to evaluating their efficacy, safety, and optimal integration into glioma management [63,64,65,66,67,68,69].

### 4.4. Limitations and Future Directions

Although this systematic literature review offers important insights, several limitations must be recognized. The studies included in the review differed significantly in terms of study design, patient populations, treatment protocols, and outcome measures, complicating efforts to conduct a meta-analysis or reach definitive conclusions. Moreover, the limited number of studies and the relatively small sample sizes in some cases highlight the necessity for larger, well-structured clinical trials.

Future directions in targeted therapies against GSC-mediated glioma progression emphasize precision medicine approaches and the development of novel molecular inhibitors [35,36,37,38,41,42,43]. Advancements in genomic and proteomic profiling can identify key mutations and signaling pathways driving GSCs, enabling the design of highly specific drugs [58,59]. Immunotherapy, particularly CAR T cell therapy, holds promise in targeting GSCs [78]. Combination therapies that integrate targeted drugs with conventional treatments like RT and CT are also being explored to overcome resistance mechanisms [49,50,51,52,53,54,55,79,80,81,82,83,84,85,86,87,88]. Moreover, leveraging artificial intelligence for drug discovery and patient-specific treatment planning is expected to enhance the efficacy and personalization of GSC-targeted therapies.

## 5. Conclusions

The intricate molecular pathways involved in GSC-mediated glioma progression offer multiple targets for therapeutic intervention, with Notch, PI3K/AKT, and Wnt/β-catenin signaling pathways being particularly critical. Targeting these pathways with specific inhibitors presents a promising strategy to disrupt GSC maintenance and tumor growth. Notch inhibitors, PI3K/AKT inhibitors, and other targeted agents have shown potential in preclinical studies, demonstrating their ability to reduce tumor growth and enhance the efficacy of existing treatments. The development of these targeted therapies, combined with advanced genomic and proteomic profiling, paves the way for personalized treatment approaches, potentially improving patient outcomes in glioma management. Continued research and clinical trials are essential to validate these findings and optimize therapeutic strategies against GSC-mediated glioma progression.

## Figures and Tables

**Table 1:** Summary of the studies included in the systematic literature review reporting on GSC-mediated glioma progression molecular mechanisms.

Author, Year	GSCs Lines	Pathway	Molecular Agent	Mechanism	Effects
Purow et al. [11] 2005	U87, U251, T98G, U373, U387, and A172	Notch	Notch-1, Delta-like-1, Jagged-1	They increase proliferation and inhibit differentiation and apoptosis in GSCs	Inhibition of differentiation and apoptosis, resulting in GBM progression
Groszer et al. [12] 2005	N/A	PTEN/PI3K/AKT	PTEN	PTEN loss causes exit from the G0/G1A (quiescent) stage of the cell cycle, and entry into the G1B and S/G2/M stages of the cell cycle	Inhibition of differentiation and increase in proliferation, resulting in GBM progression
Zagzag et al. [13] 2006	U87	HIF-1, VEGF/VEGFR	HIF-1α, VEGF	They increase the expression of CXCR4	Enhanced invasive ability, resulting in GBM progression
Piccirillo et al. [14] 2006	GBM cell lines	Smad	Smad proteins	The reduction of Smad signaling cascade increases the proliferation and inhibits the differentiation of GSCs	Inhibition of differentiation and increasing of proliferation, resulting in GBM progression
Clement et al. [15] 2007	U87	HH-GLI	GLI1	Increases self-renewal and stemness in GSCs	Inhibition of differentiation, resulting in GBM progression
Bar et al. [16] 2007	GBM cell lines	Shh	Shh ligand	Shh ligand increases the expression of Gli1	Increasing of proliferation, resulting in GBM progression
Du et al. [17] 2008	GBM cell lines	HIF-1α	SDF1α, MMP-9	HIF-1α through SDF1α increases tumor angiogenesis	Increased oncogenic potential, resulting in GBM progression
Silber et al. [18] 2008	U87 and U251	EGF, FGF	miR-124, miR-137	miR-124 and miR-137 downregulation cause inhibition of GSCs differentiation	Inhibition of differentiation, resulting in GBM progression
Gal et al. [19] 2008	GBM cell lines	SMAD	miR-451	miR-451 down-regulation inhibits GSCs differentiation	Inhibition of differentiation, resulting in GBM progression
Yeh et al. [20] 2008	GBM cell lines	NF-kβ	Leptin	Leptin induces migration and invasion of GSCs through MMP-13 production	Enhanced invasive ability, resulting GBM progression
Golding et al. [21] 2009	U87, U1242, U1242	AKT	ATM	ATM through akt controls GSCs proliferation and invasion	Enhanced invasive ability, resulting in GBM progression
Heddleston et al. [22] 2009	GBM cell lines	Notch	HIF2α	HIF2α reduces the differentiation of GSCs	Inhibition of differentiation, resulting in GBM progression
Seidel et al. [23] 2010	G55TL, G142, LN229, U87, U118, U251, U251-A, U343, U373	Notch, calcineurin	HIF-2α	Knockdown of HIF-2α eliminated the hypoxia-dependent development of the tumor stem cell phenotype	Hypoxic microenvironment contributes to GBM progression by activating an adaptive program that promotes tumor angiogenensis, invasion and survival.
Riolfi et al. [24] 2010	LN229, LN18, U138, U118	STAT 3, AKT	leptin/ObR	Leptin inhibits Rb and through STAT3, AKT increases GSC proliferation	Increasing of proliferation, resulting in GBM progression
Ernst et al. [25] 2010	GBM cell lines	Wnt/b-catenin	miR-17-92	CTGF repression caused by miR-17-92 reduces the differentiation of GSCs	Inhibition of differentiation, resulting in GBM progression
Zheng et al. [26] 2010	GBM cell lines	Wnt	PLAGL2	Enhanced PLAGL2 expression suppresses the differentiation of GSCs	Inhibition of differentiation, resulting in GBM progression
Molina et al. [27] 2010	U251	Erk, Akt	Akt	Akt activation increases tumorigenicity, stemness, and invasiveness	Increased oncogenic potential, resulting in GBM progression
Inoue et al. [28] 2010	U251	N/A	MMP-13	MMP-13 allows for GSC migration and invasion	Enhanced invasive ability of GSCs, resulting in GBM progression
Beck et al. [29] 2010	GIC3, U87	TERT-EGFR	TERT	Upregulation of EGFR by TERT plays a critical role in promoting stem cell-like features in GSCs	Persistent TERT expression in GSCs is required to maintain their undifferentiated status and resistance to drugs, resulting in progression.
Cheng et al. [30] 2011	GBM cell lines	N/A	L1CAM	High expression of LICAM promotes tumor invasion	Enhanced invasive ability of GSCs, resulting in GBM progression
Kahlert et al. [31] 2012	GBM cell lines	WNT/β-catenin	ZEB1	Wnt through ZEB1 activates epithelial-to-mesenchymal transition	Enhanced invasive ability of GSCs, resulting in GBM progression
Kaur et al. [32] 2013	GBM cell lines	Wnt/β-catenin	Wnt3a, Wnt1	Wnt3a increases cell proliferation and cell migration	Increased oncogenic potential, resulting in GBM progression
Kanno et al. [33] 2013	U87	JAK/STAT	STAT3	STAT3 allow the proliferation and self-renewal of GSCs	Increasing of proliferation, resulting in GBM progression
Carra et al. [34] 2013	GBM cell lines	pI3K/Akt, MAPK	Mcl-1	Expression of anti-apoptotic factor Mcl-1	Inhibition of apoptosis, resulting in GBM progression
Cheng et al. [35] 2013	GBM cell lines	TGF-β	SDF-1/CXCR4	The SDF-1/CXCR4 axis via TGF-β enables GSC differentiation into pericytes	Increased oncogenic potential, resulting in GBM progression
Rheinbay et al. [36] 2013	GBM cell lines	Wnt	ASCL1	ASCL1 activates Wnt signaling by repressing the negative regulator DKK1	Inhibition of differentiation, resulting in GBM progression
Gao et al. [37] 2013	U251	N/A	Fibulin-3	Fibulin-3 increases the expression of MMP-2	Enhanced invasive ability of GSCs, resulting in GBM progression
Siebzehnrubl et al. [38] 2013	GBM cell lines	ZEB1	ZEB1	ZEB1 promotes invasion by different distribution of N-cadherins on GSCs	Enhanced invasive ability of GSCs, resulting in GBM progression
Gong et al. [39] 2014	U251	PTEN/PI3K/Akt	ABCG2	ABCG2 regulates the invasion and spread of GSCs through MMP-9 activity	Enhanced invasive ability of GSCs, resulting in GBM progression
Hu et al. [40] 2016	GBM cell lines	AKT	WNT5A	WNT5A allows Endothelial Lineage Differentiation of GSC	Enhanced invasive ability of GSCs, resulting in GBM progression
Madan et al. [41] 2016	U87, U373 and GOS3	EGFR/Akt	FAT1	FAT1 through HIF1α increases the invasiveness of GSCs	Enhanced invasive ability of GSCs, resulting in GBM progression
Adamo et al. [42] 2017	U87, AM38 and U251	Wnt/β-catenin	RYK	RYK activates the WNT/β-catenin pathway and allows the cell migration	Inhibition of differentiation, resulting in GBM progression
Cenciarelli et al. [43] 2017	GBM cell lines	NOTCH, STAT3/5	Notch1	Notch1 inhibits differentiation and increases invasiveness of GSCs	Increased oncogenic potential, resulting in GBM progression
Clark et al. [44] 2017	U87	AKT	P53	AKT phosphorylation causes P53 inhibition	Increased oncogenic potential, resulting in GBM progression
Maciaczyk et al. [45] 2017	GBM cell lines	Notch	CBF1	CBF1 promotes the activation of invasive program through epithelial-to-mesenchymal transition	Enhanced invasive ability of GSCs, resulting in GBM progression
Yu et al. [46] 2017	GBM cell lines	N/A	SOX2, OLIG2, SALL2, POU3F2	These transcription factors cause GBM growth	Increase in proliferation, resulting in GBM progression
Man et al. [47] 2018	GBM cell lines	Notch	Vasorin, HIF1α/STAT3	Stabilization of Notch-1, saving it from lysosomal degradation	Inhibition of apoptosis, resulting in GBM progression
Yang et al. [48] 2018	GBM cell lines	SHH/Gli1	HDAC6	Inhibition of differentiation and apoptosis of GSCs via inactivation of SHH/Gli1	Inhibition of differentiation and apoptosis, resulting in GBM progression
Yu et al. [49] 2018	U87	FAK/Paxillin/AKT	FN	FN increases MMP-2 and MMP-9 expression and inhibits p53-mediated apoptosis	Enhanced invasive ability of GSCs, resulting in GBM progression
Shi et al. [50] 2018	GBM cell lines	STAT3	BMX	BMX activates STAT3	Increased oncogenic potential, resulting in GBM progression
Melamed et al. [51] 2018	U87	HH	Gli1	Inhibition of differentiation and apoptosis of GSCs	Inhibition of apoptosis, resulting in GBM progression
Jia et al. [52] 2018	GBM cell lines	N/A	YY1	YY1 enhances stemness in GSCs	Increased oncogenic potential, resulting in GBM progression
MacLeod et al. [53] 2019	GBM cell lines	SOX	SOCS3, USP8, DOT1L	They allow the stemness, the proliferation, and self-renewal capacity of GSCs	Increased oncogenic potential, resulting in GBM progression
Huang et al. [54] 2019	U251, U87, A172, SHG44	JAK2/STAT3	AP-2α	AP-2α downregulation inhibits the suppression of Nanog and so enhances the proliferation, migration, and invasion of GSCS	Increased oncogenic potential, resulting in GBM progression
Panza et al. [55] 2020	U87 and T98G	Notch	Leptin, Notch-1	Leptin-mediated upregulation of Notch-1 receptor and the activation of its downstream effectors	Increased oncogenic potential, resulting in GBM progression
Mitchell et al. [56] 2023	GBM cell lines	Wnt/β-catenin	WDR5	WDR5 allows the assembly of the WRAD complex and increases the expression of GSC-related oncogenic pathways.	Increased oncogenic potential, resulting in GBM progression
Jiang et al. [57] 2023	U87, U251, A172	Wnt	GSCAR (lncRNA ENSG00000250377)	GSCAR through SOX2 stabilization increases proliferation, migration, and self-renewal ability of GSCs	Increased oncogenic potential, resulting in GBM progression
Liu et al. [58] 2023	GBM cell lines	N/A	GALNT2, STAT3	GALNT2 through the expression of CD44 increases GSCs proliferation, self-renewal, and invasion	Increased oncogenic potential, resulting in GBM progression
Yun et al. [59] 2023	A172, U87, and LN229	Wnt/β-catenin	NLGN3	NLGN3 plays a role in maintaining stem cell-like properties	Increased oncogenic potential, resulting in GBM progression
Cescon et al. [60] 2023	GBM cell lines	PI3K/AKT	COL6	COL6 causes the activation of the ATR/ATM axis	Inhibition of differentiation, resulting in GBM progression
Agudelo et al. [61] 2023	GL26 and U251	N/A	HN	HN improves GSC’s capacity to induce endothelial cell migration and proliferation	Increased oncogenic potential, resulting in GBM progression
Li et al. [62] 2023	U251	N/A	FBXO7	FBXO7 controls Rbfox2-mediated splicing of mesenchymal genes	Increased oncogenic potential, resulting in GBM progression
Tao et al. [63] 2023	GBM cell lines	N/A	novel INHAT repressor (NIR)	NIR promotes ribosomal DNA (rDNA) transcription to support GSC proliferation and GBM growth	Increased oncogenic potential, resulting in GBM progression
Kahm et al. [64] 2023	U87	N/A	CTNNAL1	CTNNAL1 regulates the ability to resist RT, promote MET, secretion CCL2 that plays a role in the recruitment of immune cells to the tumor microenvironment.	Increased RT resistance and MET resulting in GBM progression
Alshahrany et al. [65] 2023	GBM cell lines	FGFR1	FGFR1	FGFR1 promotes cell migration and tumor invasion	Increased oncogenic potential, resulting in GBM progression.
Zhang et al. [66] 2023	U25, Hs683	N/A	APOBEC3	A3C expression is correlated with immune infiltration in glioma, stemness, migration, and invasion.	Increased oncogenic potential, resulting in GBM progression.
Torabidastgerdooei et al. [67] 2023	U87, U251, U118, U138	N/A	G6PC3, SLC37A4	G6PC3 and SLC37A4 upregulation is collectively associated with stemness, self-renewal capacity, and invasive properties of glioma stem cells	Increased oncogenic potential, resulting in GBM progression.
Liu et al. [68] 2023	U87, LN229	MAD2L2	MAD2L2	MAD2L2 maintains GBM stemness and promotes malignant behaviors through the regulation of c-MYC	Increased oncogenic potential, resulting in GBM progression.
Pang et al. [69] 2023	GBM cell lines	RSK4	EZH2/STAT3	RSK4 regulates the EZH2/STAT3 pathway to promote GSC maintenance and EZH2i resistance	Inhibition of differentiation and apoptosis, resulting in GBM progression
Liu et al. [70] 2023	GBM cell lines	N/A	FABP7	FABP7 upregulates SOX2, a key modulator for GBM stemness and plasticity, and ZEB1, a prominent factor in GBM MET and invasiveness	Inhibition of differentiation and apoptosis, enhanced invasive ability of GSCs, resulting in GBM progression
Xiong et al. [71] 2024	GSCs and vascular endothelial cells	IFITM3/bFGF	IFITM3	GSCs-derived IFITM3 causes activation of Jak2/STAT3 signaling and leads to secretion of bFGF into tumor environment, which results in enhanced angiogenesis	Enhanced angiogenesis resulting in GBM progression
Zhiming Fu et al. [72] 2024	GBM cell lines	N/A	SOX2	SOX2 regulates expression of genes that controls the transition to and from quiescent cell state in GBM.	Increased oncogenic potential, resulting in GBM progression.
Guo et al. [73] 2024	BG5, BG7	N/A	miR-184–3p	miR-184–3p inhibits RBM15 that activates STAT3 pathway and promotes proneural-to-mesenchymal transition	Inhibition of differentiation and apoptosis, enhanced invasive ability of GSCs, resulting in GBM progression
Maleszewska et al. [74] 2024	N/A	N/A	DMRTA2	DMRTA2 regulates gliomagenesis and tumor neovascularization	Enhanced angiogenesis resulting in GBM progression
Wang et al. [75] 2024	U87	MET–STAT3–ISG20		MET–STAT3 regulates expression of ISG20 that promotes TAM migration and M2-like polarization	ISG20-regulated macrophages promote glioma progression

Abbreviations: ABC = ATP binding cassette transporters; ATM = ataxia telangiectasia mutated;; FN = fibronectin; GBM = glioblastoma; GSCs = glioblastoma stem cells;; HIF2α = hypoxia inducible factor-2α; HN = humanin; MMP = matrix metalloproteinase; NLGN3 = synaptic proteins neuroligin 3; Shh= sonic hedgehog;; YY1 = Yin Yang 1; ZEB1 = zinc finger E-box binding homeobox 1.

## Data Availability

Data available in a publicly accessible repository.
